# The Preferential Infection of Astrocytes by Enterovirus 71 Plays a Key Role in the Viral Neurogenic Pathogenesis

**DOI:** 10.3389/fcimb.2016.00192

**Published:** 2016-12-21

**Authors:** Min Feng, Sujie Guo, Shengtao Fan, Xiaofeng Zeng, Ying Zhang, Yun Liao, Jianbin Wang, Ting Zhao, Lichun Wang, Yanchun Che, Jingjing Wang, Na Ma, Longding Liu, Lei Yue, Qihan Li

**Affiliations:** ^1^Yunnan Key Laboratory of Vaccine Research and Development on Severe Infectious Diseases, Institute of Medical Biology, Chinese Academy of Medical Sciences, Peking Union Medical CollegeKunming, China; ^2^School of Forensic Medicine, Kunming Medical UniversityKunming, China

**Keywords:** enterovirus 71 (EV71), central nervous system (CNS), astrocytes, neuron, pathogenesis

## Abstract

The pathological manifestations of fatal cases of human hand, foot, and mouth disease (HFMD) caused by enterovirus 71 (EV71) are characterized by inflammatory damage to the central nervous system (CNS). Here, the dynamic distribution of EV71 in the CNS and the subsequent pathological characteristics within different regions of neonatal rhesus macaque brain tissue were studied using a chimeric EV71 expressing green fluorescence protein. The results were compared with brain tissue obtained from the autopsies of deceased EV71-infected HFMD patients. These observations suggested that the virus was prevalent in areas around the blood vessels and nerve nuclei in the brain stem and showed a preference for astrocytes in the CNS. Interestingly, infected astrocytes within the *in vivo* and *in vitro* human and macaque systems exhibited increased expression of excitatory neurotransmitters and cytokines that also stimulated the neuronal secretion of the excitatory neurotransmitters noradrenalin and adrenalin, and this process most likely plays a role in the pathophysiological events that occur during EV71 infection.

## Introduction

Hand, foot, and mouth disease (HFMD) caused by enterovirus 71 (EV71) is an important viral infectious disease that has increasingly affected children in the Asia-Pacific region in recent years. EV71 is the pathogen primarily responsible for fatal HFMD cases (Ooi et al., 2007). Clinical pathological studies of HFMD have demonstrated that the major histopathological changes induced in severe cases of this viral infection are characterized by inflammatory damage to the central nervous system (CNS; Huang et al., 1999; Wang et al., 1999). This CNS damage selectively leads to neurogenic pulmonary edema, functional cardiopulmonary failure, and even death (Chang et al., 1998; Wu et al., 2002; Fujii et al., 2013; Yu et al., 2014). The histopathological findings in the CNS at autopsy in severe HFMD cases with EV71 infection typically include evidence of inflammatory reactions and elevated levels of cytokines in the cerebrospinal fluid (CSF) (Lin et al., 2003). However, the mechanisms by which these pathological inflammatory responses occur during this pathophysiological process and whether the interactions between EV71 and neural tissues might cause neuronal damage or interrupted homeostasis of the nervous system remain unclear. Based on general knowledge of viral encephalitis, the clinical symptoms of neurogenic injury should be associated with neuronal degeneration and necrosis (Yang et al., 2009; Ahmed et al., 2011). In cases that resemble poliomyelitis, widespread neuronal necrosis is the dominant histopathological feature associated with the replication of this virus in neurons and results in the clinical manifestation of neurogenic paralysis (Whitton et al., 2005). Although several autopsy findings have suggested that EV71 may infect neurons and cause the neuronal degeneration observed in individual fatal HFMD cases (Khong et al., 2012; Yao et al., 2012), the mechanism underlying the neuronal pathogenesis in the CNS has not been described in the literature. This situation has raised the question of how the pathological progression caused by the EV71 infection leads to the severe clinical manifestations (such as neurogenic pulmonary edema and cardiopulmonary failure) that are responsible for the high case fatality rate (Chan et al., 2000; Wu et al., 2002). The answer to this question may hold the key to understanding the pathogenesis of severe cases of EV71 infection and may require immunological and neurological analyses. Importantly, the identification of distinct mechanisms of EV71 infection with other viral encephalitis induced by various agents, even for viruses belonging to the same neurotropic virus family, might facilitate the development of efficient treatment, and prevention strategies for these diseases.

Our previous work proposed pathogenic mechanisms involved in EV71 infection in a neonatal rhesus monkey model, which was capable of capturing the essential features of the pathogenic processes involved in human EV71 infection. We found typical signs of inflammatory cell aggregation and confined vascular infiltration in the CNS together with slight degenerative modifications of certain neurons (Chen et al., 2004; Liu et al., 2011; Zhang et al., 2011). These studies also provided data that were complementary to observations in human patients. A natural next step is to investigate the effects of EV71 infection in the CNS and the host response to this virus in detail. Based on the pathological entity of inflammation with damage to a small number of neurons in CNS tissues, we focused on astrocytes as well as neurons in the present study. Astrocytes are a type of auxiliary cell that performs important supporting and regulatory functions in the CNS (Cotter et al., 2001). Thus, these cells may contribute to viral encephalitis through their important neurobiological functions in the selective regulation of neural cell activities and the modulation of synaptic transmissions (Haydon, 2000; Araque, 2006), as well as in their phagocytic capability and ability to express multiple immunologically active factors (similar to cells of the immune system) and neurotransmitters (Al-Ali and Al-Hussain, 1996; Biber et al., 2002; Barres, 2008).

In the present study, a chimeric EV71 expressing green fluorescent protein was used to observe the dynamic process and distribution of EV71 in the CNS. We subsequently examined the pathological characteristics of different regions of neonatal rhesus macaque brain tissue and compared the findings to those of autopsies of deceased EV71-infected HFMD patients diagnosed with severe neurogenic pulmonary edema and cardiopulmonary failure. Further investigations in both human and macaque *in vivo* and *in vitro* systems were performed to confirm our hypotheses that viral replication occurred in a few neurons but was mostly limited to astrocytes and that EV71 infection may increase the expression of pro-inflammatory cytokines, noradrenalin, and dopamine in astrocytes and stimulate the neuronal secretion of noradrenalin and adrenalin. Noradrenalin and adrenalin are both excitatory neurotransmitters that most likely play roles in the pathophysiological events that occur during EV71 infection.

## Materials and methods

### Ethics statement

Experiments using monkeys were designed based on the principles of “Guide for the Care and Use of Laboratory Animals” (National Research Council, 2011) and “The Guidance to Experimental Animal Welfare and Ethical Treatment” (China, 2006) and approved by the Yunnan Provincial Experimental Animal Management Association [Approval number: SCXK (Dian) 2011-0005] and the Experimental Animal Ethics Committee of the Institute (Approval number: YISHENGLUNZI [2011] 15). All of the experimental protocols involving human samples were approved by the Human Ethical Commission at Kunming Medical University. All of the patients' parental or legal guardians provided written informed consent.

Each newborn monkey was housed in a single cage with its mother in a large room (BSL-2 conditions) with sufficient fresh air and natural light, thus permitting visual, olfactory, and auditory interactions with other animals. The room temperature was maintained at ~22–24°C during the experiments. Food and water were readily available. The animals were provided access to environmental enrichment (such as approved toys) to promote psychological well-being. Anesthesia was performed when needed during the experimental procedure. All animals were fully under the care of veterinarians at the Institute of Medical Biology, Chinese Academy of Medical Science (CAMS).

### Human tissues

Tissue samples from six deceased patients who were confirmed to be infected with EV71 were obtained from the School of Forensic Medicine, Kunming Medical University, fixed in formalin, and embedded in paraffin. The average age of the 6 cases was 23.1 months (range 9–42 months), and all cases were male. Two other brain samples from children without encephalitis (average age 13.5 months, both males) were used as negative controls.

### Viruses and cells

Two EV71 viruses were used in this study. The first, FY-23 (GenBank: EU812515), was isolated from the respiratory tract secretions of a child with severe cardiopulmonary collapse in Fuyang, China, in May 2008 (Wang et al., 2010). The second virus was an EV71 chimera expressing enhanced green fluorescent protein (EGFP-EV71). The details of this virus were descripted in another our manuscript which is under view (Zhao et al., Dynamic interaction of enterovirus 71 with dendritic cells in infected neonatal rhesus macaques). Briefly, the first and second fragment of the EV71 virus (FY-23) genome was amplified by RT-PCR and cloned into the Psp64 vector successively, leading to the construction of clones containing the complete EV71 genome. Then nucleotides encoding EGFP were inserted next to the start codon of the open reading frame in the complete EV71 genome clone. A recombinant plasmid carrying EGFP was linearized and used as a template for in vitro transcription. Complete RNA transcripts were transfected into Vero cells and then typical CPEs and green fluorescence were observed, EV71 chimera expressing enhanced green fluorescent protein (EGFP-EV71) was harvested using low-speed centrifugation (4°C, 10 min, 2000 × g), and the supernatants which include EGFP-EV71 were stored. These strains were grown in Vero cells (American Type Culture Collection, Manassas, VA, USA). The virus was harvested and frozen at −80°C following the development of typical cytopathic effects (CPE) at a concentration of 10^6.0^–10^7.0^ CCID_50_/ml. Human neurons and astrocytes (originating from the cerebrum, brain stem, and hippocampus, Cat. No. 1800, 1830, 1840, 1520, 1560) were purchased from ScienCell Research Laboratories (San Diego, CA, USA) and cultured in neuronal medium and astrocyte medium, respectively (Cat. No. 1521 & 1801, ScienCell). These commercial cells were characterized based on the corresponding specific markers according to the instructions of ScienCell. The rhesus macaque astrocytes were separated from the cortex of the animal, purified, and cultured as previously described (Yue et al., 2013). The human diploid cell line KMB 17 (Guo et al., 1981) was maintained in DMEM (Dulbecco's modified Eagle's medium; Thermo Scientific, Beijing, China) containing 8.0% fetal bovine serum (FBS) (GIBCO, Grand Island, NY, USA) and grown to confluence prior to use.

### Infection of EV71 in rhesus macaque *in vivo*

Eight monkeys were infected with EV71 (10^4.5^CCID_50_/monkey) via the respiratory tract by tracheoscopy or intracerebellar injection according to standard protocols (12, 13) under anesthesia. An additional two monkeys were treated with saline as negative controls. All animals were housed in isolation for 2 weeks before the initiation of the study. A neutralization test was conducted to confirm that they did not have antibodies against EV71 prior to experimental infection. The infected neonates were breast-fed by female monkeys. The pathogenic and histopathological examinations were performed on brain tissues of neonates sacrificed on days 3 and 7 p.i. under anesthesia. Venous blood samples were collected in EDTA-coated capillary tubes.

### Infection of EV71 in neurons and astrocytes *in vitro*

Human neurons and astrocytes were infected with EGFP-EV71 (multiplicity of infection [MOI] = 0.05) for 60 min at 37°C and washed twice with PBS. Then, 3% FBS was added prior to continuous incubation. The samples were harvested at different time points and stored at −80°C prior to analysis.

Alternatively, human and macaque astrocytes were infected with EV71 (FY-23) (multiplicity of infection [MOI] = 0.05 or 0.1) for 60 min at 37°C and washed twice with PBS. Then, 3% FBS was added prior to continuous incubation. The samples were harvested at different time points and stored at −80°C prior to analysis. Human astrocytes in the upper layer and human neurons in the lower layer were co-cultured in a double layer co-culture system (Millipore, Bedford, MA, USA). The experiment of infection of EV71 in neurons has been repeated twice, and in astrocytes has been repeated three times.

### Virus titration

Virus titration was performed using a microtitration assay according to a standard protocol (Arita et al., 2008). Briefly, serial 10-fold dilutions were generated from the virus stocks and then added to 96-well plates coated with Vero cells and incubated at 37°C in 5% CO_2_. The presence of CPE was recorded 7 days p.i.

### RNA extraction and quantitative real-time PCR (qRT-PCR)

Viral RNA was extracted from EV71-infected neurons or astrocytes using the Mino Best Viral RNA/DNA Extraction Kit (Takara, Japan) according to the manufacturer's instructions. qRT-PCR amplification was performed using the Taqman One-Step RT-PCR Master Mix (Takara, Japan) on a 7500 Fast RT-PCR system (Applied Biosystems, Foster City, CA, USA) as previously described (Liu et al., 2011). For C3 expression analysis, total RNA was extracted from EV71-infected human astrocytes using the TRIzol reagent (Tiangen Biotech, China) according to the manufacturer's instructions. The PCR primers and TaqMan™ probes used to amplify complement C3 and the housekeeping gene β-actin were employed using a previously described protocol (Serinsöz et al., 2005). The relative C3 mRNA expression levels were calculated using the deltaCT method. The relative mRNA expression of the negative controls was given an arbitrary value of one for comparison purposes, and the mRNA expression of EV71-infected astrocytes was expressed relative to the negative control astrocytes. The sequences of the EV71-specific primers used to detect the negative-stranded RNA genomeswere as follows: forward primer, 5′-ccaccgatgatggcgtttc-3′ and reverse primer, 5′-ttccctgcttgtgctgagac-3′.

### Histopathology, immunohistochemistry, and scoring

Tissue samples were fixed in 4% formalin in PBS, dehydrated in a graded ethanol series and embedded in paraffin before obtaining 4-μm sections for the experiments, which included hematoxylin and eosin (HE) staining and immunohistochemistry assays. The EV71 antigen was detected in a standard immunoperoxidase procedure using a mouse anti-EV71 monoclonal antibody (Clone 10F0; Abcam, Cambridge, MA, USA) and horseradish peroxidase (HRP)-conjugated anti-mouse IgG antibodies (Sigma, Deisenhofen, Germany), followed by color development with diaminobenzidine for the detection of the antigen-antibody reaction. To detect the percentages of EV71 infected neurons and astrocytes in the brain stem from macaques and human patients, neurons and astrocytes were counted from a total of 20 and 30 sections from 4 animals and 6 patients (five sections of each subject), respectively.

### Immunofluorescence assays

To detect the green fluorescent signals of EGFP-EV71virus, frozen tissue sections of rhesus monkeys were sectioned using a cryostat microtome (CM1950; Leica) according to the manufacturer's protocols. Frozen sections were observed under fluorescence microscopy (DMI6000B; Leica). The EV71 antigen in paraffin sections of human tissue was detected using a primary mouse anti-enterovirus 71 monoclonal antibody (Chemicon, Temecula, CA, USA) and a secondary Alexa Fluor 488 donkey anti-mouse IgG antibody (Life Technologies, Thermo Fisher Scientific Inc.). Neurons and astrocytes were detected using a rabbit anti-NeuN antibody and a rabbit anti-GFAP antibody (Abcam Ltd., Cambridge, UK) as the primary antibodies, respectively, and an Alexa Fluor 594-conjugated donkey anti-rabbit IgG antibody (Life Technologies, Thermo Fisher Scientific Inc.) as the secondary antibody. The cell nucleus was detected using DAPI. The staining procedure was performed according to a standard protocol (Laszik et al., 1996; Jiang et al., 2004; Liu et al., 2011). The immunofluorescence assay was performed with a Nikon C1 laser scanning confocal microscopy (LSCM) system (Nikon, Japan).

### Cytokine and monoamine analysis

The cytokine levels in the culture supernatants and homogenized brain samples were evaluated using ELISA kits (Neobioscience Technology CO., Ltd.). An aliquot of the harvested culture supernatants was analyzed to determine the levels of adrenalin, noradrenalin, and dopamine by monoamine analysis with a 3-CAT RIA kit (DIAsource, Louvain-la-Neuve, Belgium).

### Transmission electron microscopy (TEM) observations

EV71-infected astrocytes were harvested and observed by TEM as previously described (Yue et al., 2013). Briefly, the samples were fixed, washed three times, dehydrated, embedded, stained, and observed. This experiment has been repeated twice.

### Infection inhibition assays

Human and rhesus macaque astrocytes were pre-incubated for 1 h at 37°C with the following antibodies (50 μg/ml) against EV71 receptors: anti-CD162/PSGL-1 (BD Biosciences, San Jose, CA, USA), anti-annexinA2 (Abcam, Cambridge, UK), anti-DC-SIGN (Abcam), anti-SCARB2/LIMP2 (Novus Biologicals, Littleton, CO, USA), anti-FGF receptor 3 (FGFR3; Abcam), anti-CCR5 (GeneTex, San Antonio, TX, USA), and anti-CCR3 (GeneTex). The cells were then infected with EV71 (MOI = 0.05), washed twice with PBS and collected at different time points p.i. to determine the viral loads. This experiment has been repeated twice.

In the experiment using human purified complement, a low concentration of virus corresponding to an MOI of 0.005 was co-incubated with human purified complement (Cat. No. 204881, Merck Millipore) at concentrations of 5, 25, and 50 μg/ml (based on the physiological concentration in brain tissue; Barnum, 1995; Jongen et al., 2000) or control buffer for 1 h, and then these mixtures were added to human astrocyte cultures for the observation of CPE. This experiment has been repeated twice.

### Statistical analysis

All of the data were expressed as the mean + SD. The differences between two groups were evaluated using an independent sample *t*-test. For all of the comparisons, a significant difference was assumed at *p* ≤ 0.05; all of the data were analyzed using SPSS version 19.

## Results

### Dynamic distribution of EV71 in the CNS of infected rhesus monkeys

**Figure 1:**
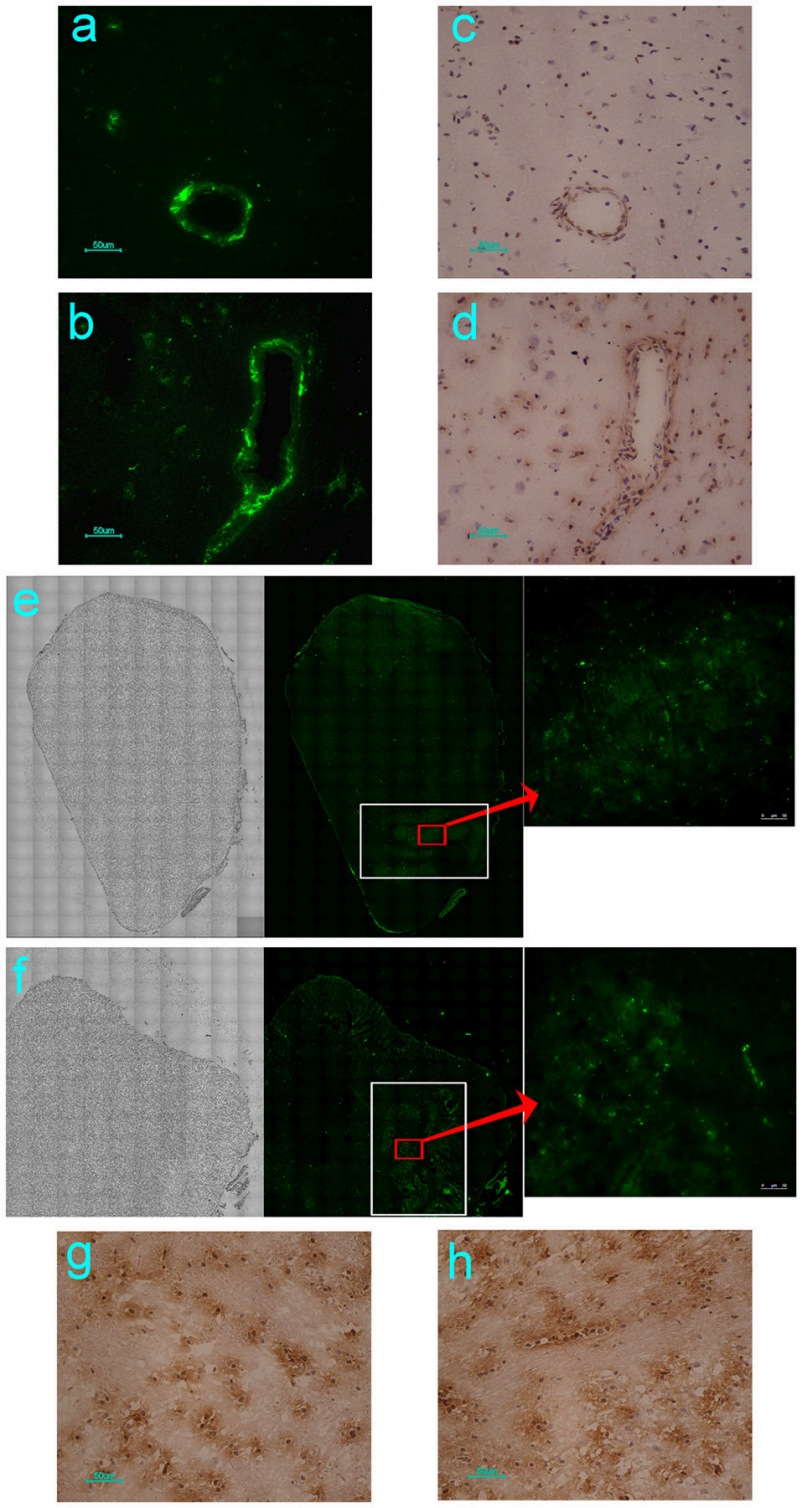
**Dynamic distribution of EV71 in the CNS of infected rhesus macaques**. The green fluorescent signal and EV71 antigen were aggregated around the blood vessels on day 3 **(A,C)** and distributed in the tissues of the brain stem on day 7 after EGFP-EV71 infection **(B,D)**. The green fluorescent signal was detected and the EV71 antigens were expressed (red rectangle) in olivary nuclei (white rectangle) on day 7 after EGFP-EV71 infection via the respiratory tract **(E,G)** and cerebral injection **(F,H)**. The images are shown at 100 × magnification in e and f and were combined automatically using Leica AF Multi-Dimensional Acquisition software.

Our previous study using neonatal rhesus monkeys provided details concerning the pathological process of EV71 infection in this animal model (Liu et al., 2011). The virus infected the animals via the respiratory tract and resulted in typical clinical manifestations similar to those observed in human patients, including fever, and the appearance of vesicles in the mouth and limbs (Liu et al., 2011). The histopathological detection provided pathological data for these clinical symptoms (Liu et al., 2011; Zhang et al., 2011). Based on these results, the work described here used this rhesus model to investigate the dynamic process and distribution of EV71 virus infection in the CNS. A chimeric EGFP-EV71 strain capable of expressing a green fluorescent signal during viral replication was used to infect macaques through the respiratory tract and cerebral injection. The macaques were infected with the virus and sacrificed by anesthesia on days 3 and 7 post-infection (p.i.), and then different regions of the macaque CNS tissue were observed by fluorescence microscopy. First, the results of both EGFP-EV71 infection routes suggested that the green fluorescent signal was aggregated around the blood vessels on day 3 p.i., and distributed in the tissues on day 7 p.i., especially in the brain stem (Figures 1A,B). Parallel immunohistochemistry observations using an antibody directed against an EV71 antigen showed consistent results, with the viral antigen aggregated in the areas around the blood vessels and sporadically expressed in other areas (Figures 1C,D). Second, the distribution of the fluorescence signal in the coronal plane of the brain stem suggested that EV71 preferred the inferior olivary nuclei in animals infected via either the respiratory tract or cerebral injection (Figures 1E,F). The same results were obtained via immunohistochemical detection (Figures 1G,H).

### Virus distribution and histopathological characteristics in CNS tissue from infected macaques and humans

**Figure 2:**
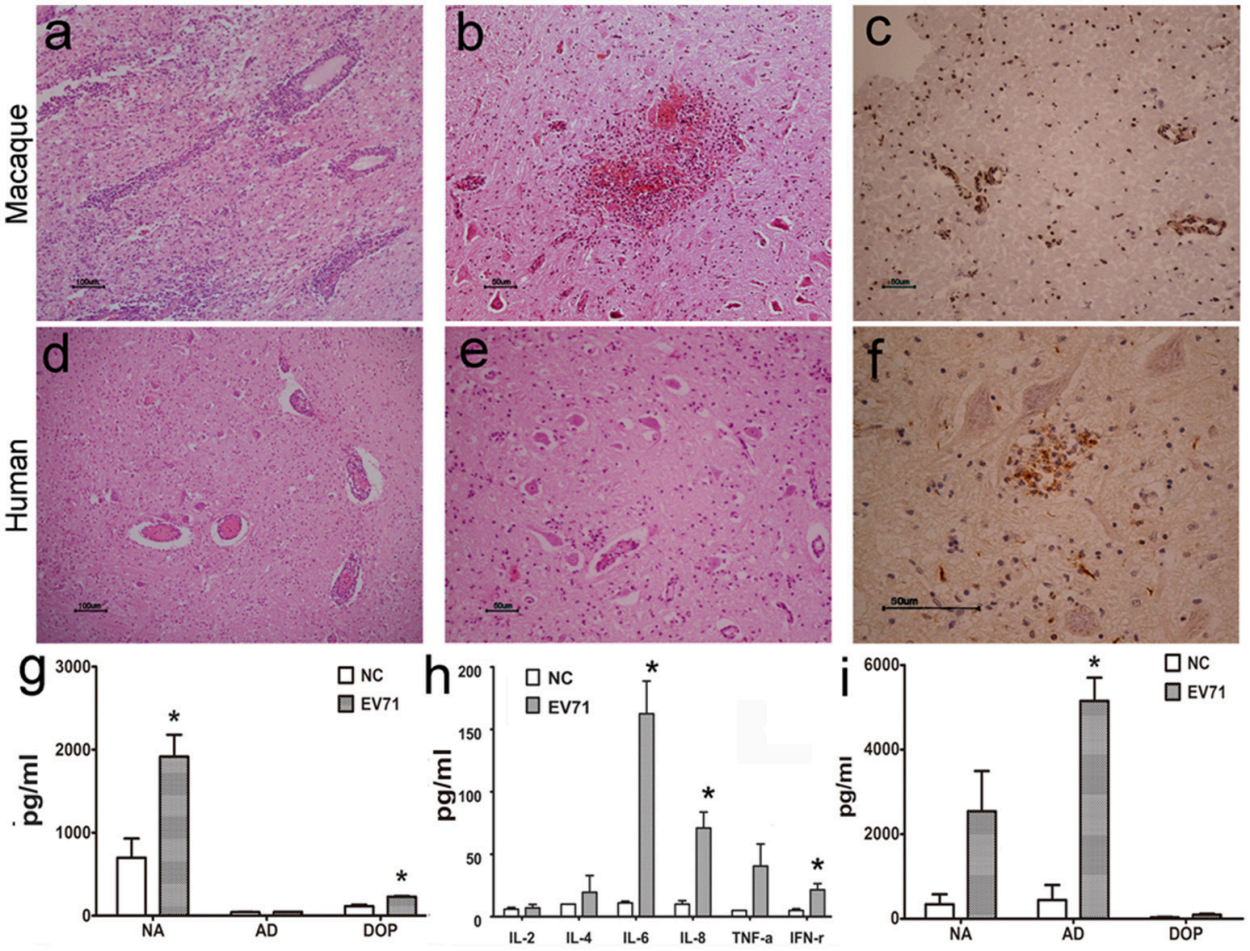
**Histopathological characteristics of CNS tissue from EV71-infected rhesus macaques and human patients**. Typical pathological changes in the brain stem include the following: **(A,D)** inflammatory cells aggregated near vascular tissue; **(B,E)** neuron degeneration and glial cell proliferation and glial nodule formation; **(C,F)** EV71 viral antigen expression in CNS tissues from EV71-infected rhesus macaque and human patients. Monoamine **(G)** and cytokine **(H)** detection in brain stem homogenates, and monoamine **(I)** detection in the peripheral blood from infected rhesus macaques. NA, noradrenalin; AD, adrenalin; DOP, dopamine; NC, negative control. ^*^p ≤ 0.05 compared with the corresponding control group.

Typically, viral proliferation in tissues is capable of inducing a host inflammatory response, even in the CNS (Lin et al., 2003). Thus, the infiltration of inflammatory cells, hyperemia, edema and tissue necrosis were proposed as evidence of viral infection. Here, the histopathological assessment of different regions of the brain from animals infected either via the respiratory tract or cerebral injection provided supportive data to further our understanding of the dynamic virus distribution in the CNS. The pathological changes in the brain stem were characterized as (1) typical infiltration confined to the vasculature by inflammatory cells and an injured blood vessel structure (Figure 2A); (2) sporadic neurons showing degeneration and necrosis (Figure 2B); and (3) more glial cells expressing EV71 antigen than neurons in areas around the blood vessels and the inferior olivary nuclei (Figure 2C). These pathological changes in the brain stem were similar to those found during autopsies of EV71-infected HFMD patients (Figures 2D–F). Similar pathological changes were observed in other brain regions, including the cerebrum and cerebellum, of EV71-infected macaques and humans. In addition, perivascular infiltration, fewer degenerated neurons and more infected glial cells were common characteristics of the viral infection in both macaques and humans. Approximately 16 and 20% of the 2500 neurons in the brain stem examined in a total of 20 sections from four infected macaques and 30 sections from six human samples, respectively, exhibited evidence of neuronal degeneration, including shrinkage, neuronophagia, and nuclear disappearance. The analysis of homogenized brain stem tissue demonstrated the up-regulation of certain neurotransmitters, especially noradrenalin and dopamine (*ps* < 0.037, compared with corresponding negative control groups) (Figure 2G) and cytokines, such as IL-6, IL-8, and IFN-γ (*ps* < 0.046) (Figure 2H), in addiation, an obvious up-regulation of adrenalin in the peripheral blood of infected macaques (*p* = 0.005) (Figure 2I).

### EV71 prefers to proliferate in astrocytes instead of neurons

**Figure 3:**
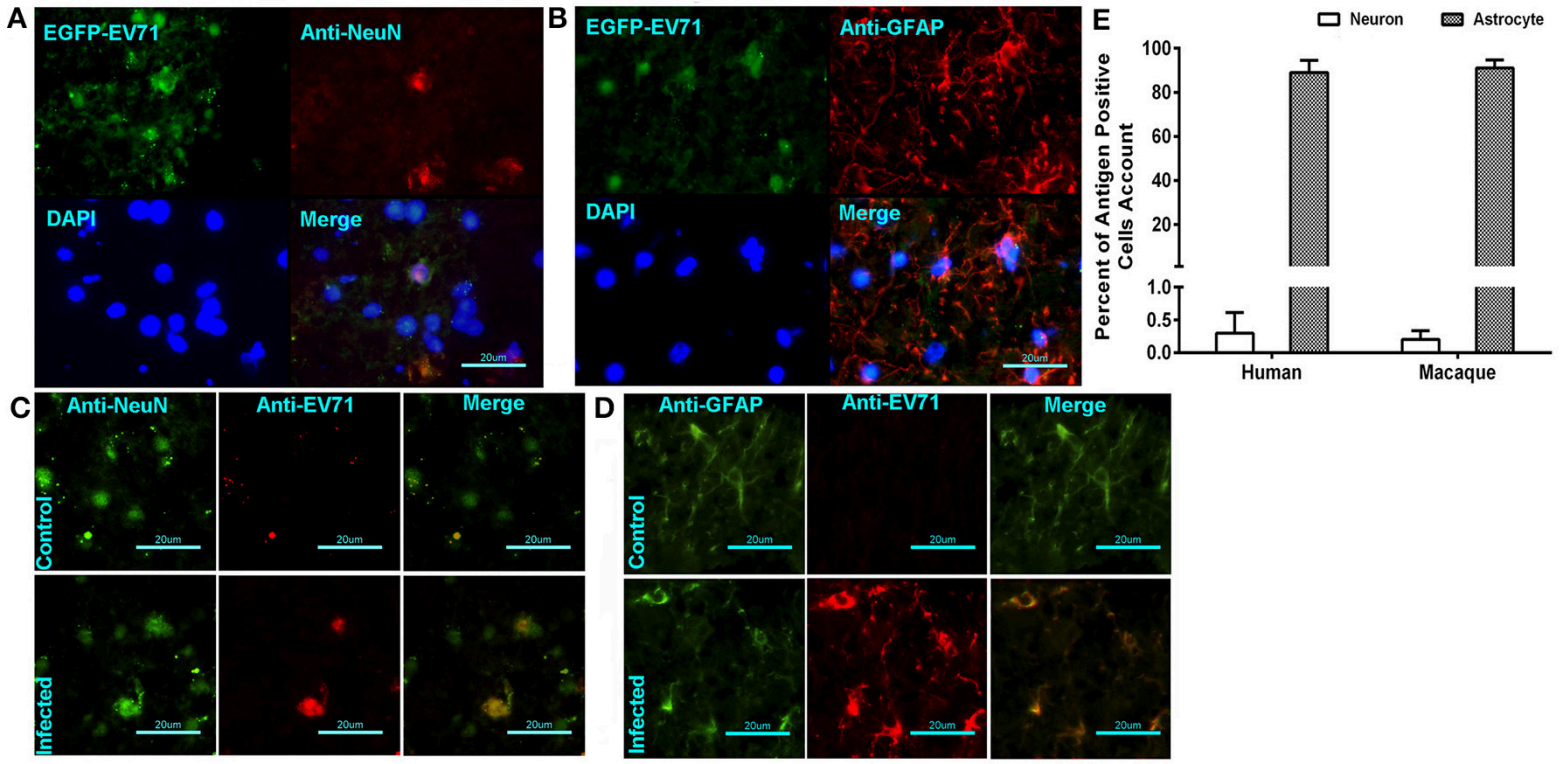
**EV71 was detected in neurons and astrocytes in CNS tissues from infected rhesus macaques and human patients**. The green fluorescent signal of EV71 was detected in neurons **(A)** and astrocytes **(B)** labeled with red fluorescence by an anti-neuron antibody and an anti-GAFP antibody, respectively. **(B)** Immunofluorescence confocal microscopy observations of neurons **(C)** or astrocytes **(D)** and EV71 antigen in CNS tissues from infected human patients. **(E)** The percentages of antigen-positive neurons and astrocytes in the brain stem (from 20 sections from 4 animals and 30 sections from 6 patients).

In our previous work using the EV71-infected macaque model, viral replication in the brain tissue was revealed through the dynamic growth curve based on qRT-PCR and virus titration (Zhang et al., 2011). This finding clearly suggested that cells in the brain tissue were targeted by the virus. Our work here identified the infected cells that supported viral proliferation based on fluorescent signal detection and immunohistochemical staining. The green fluorescent signal was co-localized not only with the red fluorescence-labeled anti-neuron antibody (Figure 3A) but also with the red fluorescence-labeled anti-GAFP antibody that targets astrocytes (Figure 3B). A higher rate of infected astrocytes and lower rate of infected neurons were noted in the areas around vessels and the inferior olivary nuclei. Similar findings were observed in human brain stem (Figures 3C,D). These results figured out that EV71 in CNS showed the preference for astrocytes than neurons with an unknown reason. For further identification of this characterized phenomenon, the separate observations of the brain stem from infected macaques 7 days p.i. and human patients showed that 0.25% and 0.32% of the neurons from a total of 20 and 30 sections from four animals and six patients (five sections of each subject), respectively, were antigen positive, whereas ~91 and 89% of the astrocytes were antigen positive, respectively (Figure 3E).

### Analysis of EV71 replication in human neurons and astrocytes in an *in vitro* culture system

**Figure 4:**
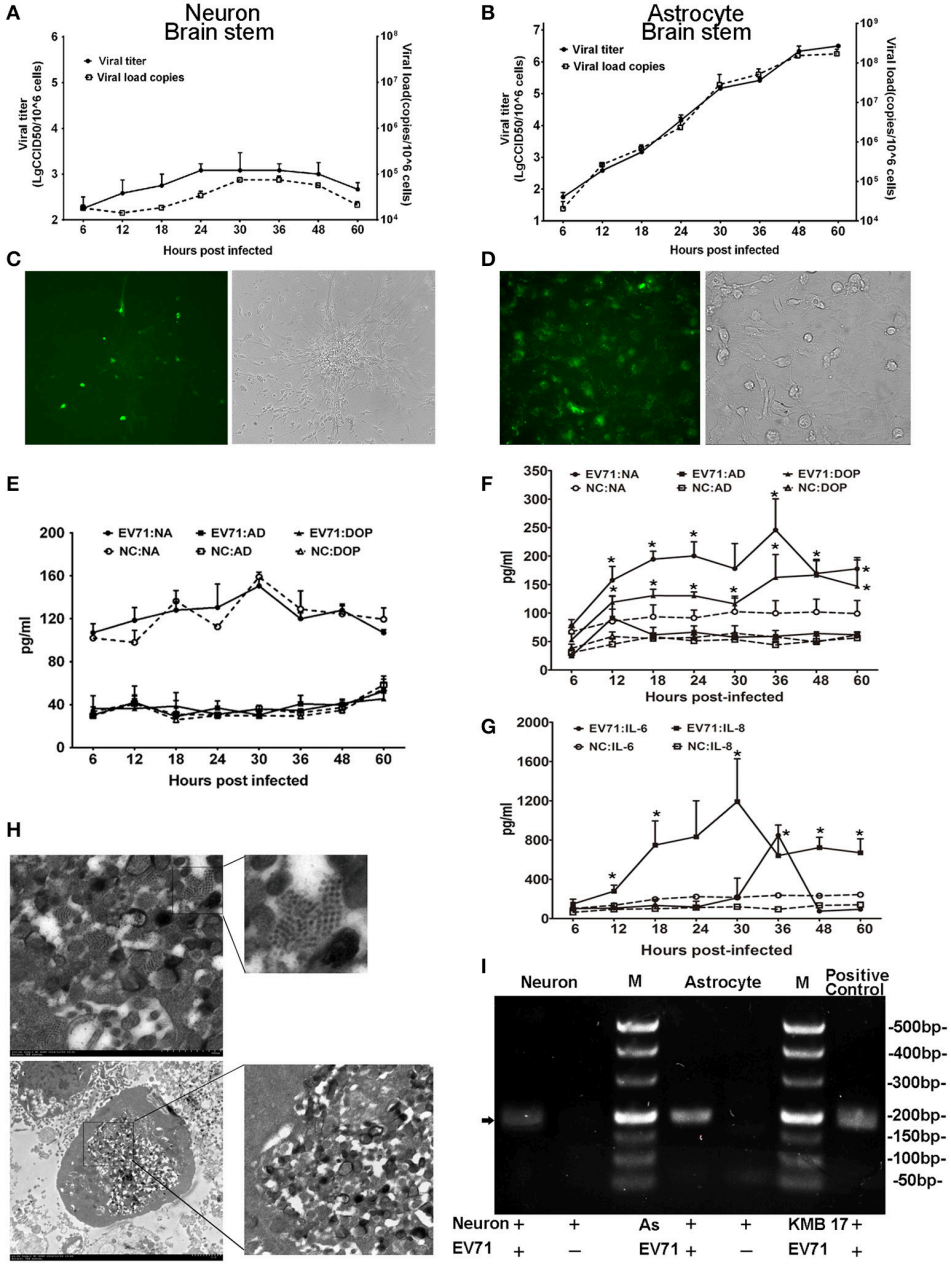
**Detection of EV71 replication in cultured neurons and astrocytes *in vitro***. Proliferation of the virus in cultured human neurons **(A)** and astrocytes **(B)** was measured based on virus titration and the viral load from 6 to 60 h p.i. Immunofluorescence microscopy observations of the EV71 antigen in cultured EV71-infected neurons **(C)** images are shown at 100 × magnification) and astrocytes **(D)** images are shown at 400 × magnification.). Monoamine release by neurons **(E)** and astrocytes **(F)** from 6 to 60 h p.i. Cytokines released by astrocytes **(G)** between 6 and 60 h p.i. The cells were infected with EV71 (MOI = 0.05). NA, noradrenalin; AD, adrenalin; DOP, dopamine; NC, negative control. ^*^p ≤ 0.05, compared with the corresponding control group. **(H)** A characteristic lattice-like arrangement of viral particles was observed in human astrocytes induced by EV71 infection as observed by TEM. Astrocytes were infected with EV71 (MOI = 0.1), and samples were collected at 24 h p.i. **(I)** Presence of negative-stranded RNA genomes in the cells is shown (black arrow). M, marker; AS, astrocyte.

To investigate the potential roles of EV71 in neurons and astrocytes (either alone or combination) in the CNS, we cultured human neurons isolated from the cerebrum and brain stem to examine EV71 replication in this cell type. The neurons were infected with EGFP-EV71 at an MOI of 0.05. The results of these two infected neuronal cultures isolated from the brain stem (Figure 4) and cerebrum (Figure S1) are similar. The viral kinetic growth curve showed a low peak within 60 h p.i., which was consistent with the detection results by qRT-PCR (Figure 4A) and fluorescence observations (Figure 4C). Morphological observations also indicated that the EV71-infected neurons maintained their structural and morphological characteristics (Figure 4C), which was most likely because of the low infection efficiency. Therefore, positive TEM results for neurons were not available. Next, the supernatants of these two infected neuronal cultures were evaluated to detect the expression of the neurotransmitters noradrenalin, adrenalin, and dopamine. The results showed that the expression values were equivalent to those of the uninfected negative control (Figure 4C and Figure S1).

Furthermore, human astrocytes originating from the cerebrum, brain stem and hippocampus were also used to examine EV71 replication. Three astrocyte cultures were infected with EV71 at an MOI of 0.05. The results of these three infected astrocytes isolated from the brain stem (Figure 4), cerebrum and hippocampus (Figure S2) are similar. A typical virus kinetic growth curve was observed with a titer peak within 48–60 h p.i., which was consistent with the trend observed by qRT-PCR (Figure 4B). A morphological analysis of these infected astrocytes revealed typical cytopathic effects (CPE) including swelling, rounding, enhanced refraction and eventually disruption. Notably, the green fluorescence emitted from the virus was detected in the astrocytes by fluorescence microscopy (Figure 4D). Concomitantly, TEM images of these infected astrocytes revealed that the internal structures of the cytopathic cells were no longer discernable ~24 h p.i., (Figure 4H). A variety of polymorphous double membranes were visible in the cytoplasm, including a multi-layered concentric membrane structure similar to that observed in cells infected with poliovirus (Egger et al., 2000) and a lattice-like virus particle embedded in and flanked by the endoplasmic reticulum membrane. Additionally, we probed for negative-stranded viral RNA in the neurons and astrocytes to confirm viral replication and found that EV71 was capable of proliferating in neurons with a lower efficiency relative to astrocytes (Figure 4I). Neurotransmitters and cytokines were measured in the supernatants of these infected astrocytes because these cells types function in the modulation of the nervous system and the immune response in the CNS (Qiu et al., 1996; Wolf et al., 2008; Singhal et al., 2014). The results showed obviously up-regulated levels of noradrenalin and dopamine (*ps* < 0.028, compared with corresponding negative control groups) (Figure 4F) as well as IL-6 and IL-8 (*ps* < 0.046, compared with corresponding negative control groups) (Figure 4G). Experimental observations of the viral infection in astrocytes from a neonatal macaque cultured *in vitro* presented similar results, with neurotransmitters (e.g., noradrenalin) and cytokines (e.g., IL-6 and IL-8) showing higher expression (*ps* < 0.036, compared with corresponding negative control groups) that was consistent with the results from human astrocytes (Figure S3).

### Engulfment of EV71 by astrocytes facilitates viral replication

**Figure 5:**
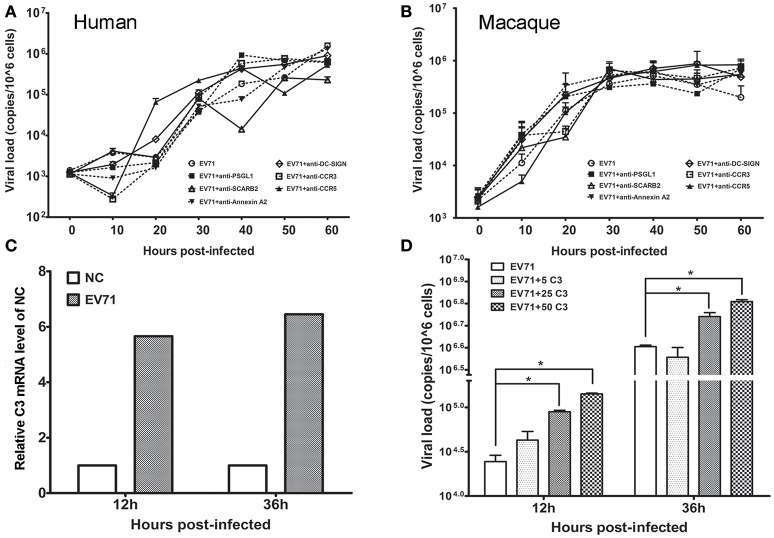
**Effect of astrocyte engulfment on EV71 infection in astrocytes**. Antibodies against EV71-specific receptors failed to inhibit EV71 infection in human **(A)** and macaque astrocytes **(B). (C)** Detection of complement C3 mRNA expression in infected human astrocytes. NC: negative control. **(D)** Incubation of complement and virus facilitates viral replication in human astrocytes, with a peak at 12–36 h. The viral loads of the infected astrocytes and relative expression of complement mRNA were measured using TaqMan-based qRT-PCR. β-actin was used as the house keeping gene.

Based on the *in vivo* and *in vitro* detection of EV71 infection in human neurons and astrocytes using the methods described above, it was reasonable to speculate that astrocytes were a main target for EV71 infection *in vivo* after the virus entered the human and macaque CNS during clinical infection. However, specific antibodies against known EV71 receptors, including SCARB-2, PSGL-1, and DC-SIGN, frequently failed to inhibit viral replication in human (Figure 5A) and macaque astrocytes (Figure 5B). Based on our knowledge of the basic phagocytic functions of astrocytes and the role of complement factors produced in astrocytes during this process (Al-Ali and Al-Hussain, 1996; Jongen et al., 2000; Barres, 2008), EV71 infection of astrocytes was, therefore presumed to be directly attributable to the phagocytosis of virus particles. To confirm this hypothesis, the expression of complement mRNA in infected human astrocytes was detected and the role of human complement in astrocyte infection was investigated. The increased expression of complement mRNA was observed in infected astrocytes compared with the control cells in the first experiment (Figure 5C). In the second experiment, a low titration of virus corresponding to an MOI of 0.005 was co-incubated with purified human complement at a concentration of 5 μg/ml (a physiological concentration in brain tissue, Barnum, 1995; Jongen et al., 2000), 25 or 50 μg/ml and control buffer for 1 h, and these mixtures were then added to the astrocyte cultures for observation of CPE. The results showed that the incubation of virus with complement expedited viral replication in human astrocytes, which peaked at least 12–36 h earlier than that in control cells (*ps* < 0.025, compared with corresponding negative control groups) (Figure 5D).

### Interaction of infected astrocytes and neurons in a culture system

**Figure 6:**
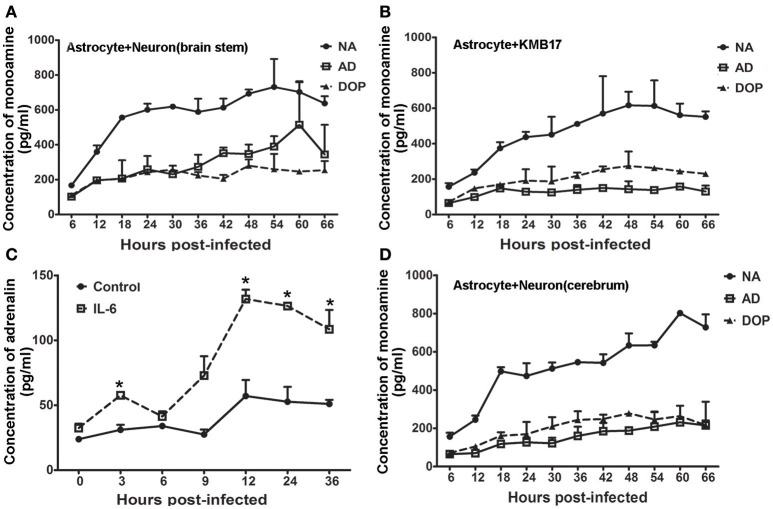
**Modulated IL-6 release by infected astrocytes induces adrenalin secretion**. Monoamine release by neurons from the brain stem **(A)** or KMB 17 cells **(B)** co-cultured with EV71-infected astrocytes from the brain stem. **(C)** Adrenalin release by neurons from the brain stem treated with IL-6 (15 ng/ml). **(D)** Monoamine release by neurons from the cerebrum co-cultured with EV71-infected astrocytes from the brain stem. Astrocytes were infected with EV71 (MOI = 0.05). The cultured cell supernatant samples were collected at different hours p.i. ^*^p ≤ 0.05, compared with the corresponding control group. NA, noradrenalin; AD, adrenalin; DOP, dopamine.

Astrocytes were previously demonstrated to have the capacity to modify the functions of individual neurons in the CNS. Astrocytes serve as a type of supportive cell and play a role in the encephalic inflammatory response (Mucke and Eddleston, 1993). Based on greater up-regulation of IL-8 compared with IL-6 in astrocytes from the brain stem and the secretion of a higher level of IL-6 by astrocytes from the cerebrum and hippocampus (Figure 4G and Figure S2) than from the cerebellum, we designed a double-layer co-culture system consisting of an upper layer of human astrocytes from thebrain stem and a lower layer of human neurons isolated from the cerebrum and brain stem. We used this system to investigate the potential interaction of astrocytes and neurons during EV71 infection. A double-layer co-culture system containing human astrocytes from the brain stem and human fibroblasts was used as a control. The experiment was initiated with the infection of the astrocytes with the virus at an MOI of 0.05, followed by the detection of neurotransmitters in the supernatant of the neurons cultured in the lower layer at various time points. Interestingly, the experimental results of the neurons isolated from brain stem showed that adrenalin increased gradually and slightly after it was first detected at 6 h p.i., and this increase was significant compared with the control at up to 66 h p.i., but noradrenalin and dopamine originating from the neurons were unchanged compared with the baseline levels secreted by astrocytes during this infection (Figure 6A). This increased levels of adrenalin from the neuron of the brain stem in this co-culture system suggested that increased neurotransmitter expression occurred after 36 h p.i., compared with the infected astrocyte control (Figure 6B). To confirm this increase in adrenalin, we added noradrenalin, IL-6 and IL-8 at the amounts measured in the supernatant of infected astrocytes at 36–48 h p.i., as described in Figures 4F,G to the cultured neurons. The results indicated that IL-6 induced a slight up-regulation of adrenalin (*ps* < 0.033, compared with corresponding negative control groups) (Figure 6C), whereas noradrenalin and IL-8 had no effect (Figure S4). However, the levels of noradrenalin produced by the neurons isolated from the cerebrum co-incubated with infected astrocytes showed much weaker up-regulation compared with that of neurons from the brain stem (Figure 6D). Strikingly, no change in neurotransmitter secretion was detected in the double-layer co-culture system that consisted of EV71-infected astrocytes and human fibroblasts compared with the typical CPE clearly observed in the human fibroblasts at 24 h p.i., (Figure S5).

## Discussion

The present study was designed to trace the EV71 virus and to identify the relationship between pathological characteristics and viral pathogenesis during EV71 infection. We first described the dynamic distribution of EV71, and the observations suggested that the virus was prevalent in areas around the blood vessels and nerve nuclei in the brain stem. The entry of EV71 into the CNS, except via retrograde axonal transmission along the peripheral nerve, most likely occurs through the blood-brain barrier in a specific manner based on the results from necropsies of macaque infected with a chimeric EV71 strain expressing the green fluorescent protein as well as autopsies of human EV71-infected HFMD patients. Our observations in this study provide a conceptual framework for the pathophysiological process underlying EV71 infection in the CNS, which led us to hypothesize that infected astrocytes may play a major role in the pathogenesis of neurogenic pulmonary edema and cardiopulmonary failure.

Astrocytes are the most abundant glial cell in the CNS and possess a variety of functions, including the selective provision of essential assistance to neurons, formation of the blood-brain barrier (Haydon, 2000; Cotter et al., 2001; Araque, 2006), and modulation of neuronal functions based on specific physiological capabilities (Mucke and Eddleston, 1993), such as their ability to engulf invading pathogens and release active factors. Moreover, astrocytes are important for the maintenance of CNS homeostasis (Biber et al., 2002). This study confirmed that EV71 not only infects neurons of the cerebrum and brain stem but also possesses an obvious preference for astrocytes from either the cerebrum or the brain stem and hippocampal areas. Our data collected through *in vivo* and *in vitro* experiments provided comprehensive details on viral proliferation in astrocytes and indicated that EV71 has a stronger tendency to replicate in astrocytes than in neurons. The experiments also suggested that viral infection in these cells was dependent on phagocytosis and the presence of complement in at least the *in vitro* culture system, which suggests that the virus (which is an exogenous pathogen facing astrocyte immune cells in the CNS) exhibits a preference for astrocytes compared with neurons, a finding that facilitated the elucidation of the pathogenesis of EV71 infection in the CNS. Our previous data and the results reported by others indicated that EV71 is capable of transferring to target organs in the body by sojourning in immune cells, such as CD14^+^or dendritic cells, and most likely spreads to the CNS through peripheral nerves via retrograde axonal transport (Wang et al., 2013). We observed aggregated virus in the cells of the blood-brain barrier structure during the early phase of infection (day 3 p.i.), which was different than the peak viral loads observed in the peripheral nerves and CNS of rhesus monkeys on days 7 and 10, respectively (Wang et al., 2013). In this case, we speculated that EV71 circulated to the blood-brain barrier structure and encountered astrocytes when it sojourned in immune cells, such as DCs. Because the virus demonstrated a preference for astrocytes, this step would have created a relay for viral proliferation and enabled the viral progeny to transfer into the CNS. This process would explain the high viral load in CNS tissues during viral infection and the subsequent severe inflammatory reaction in the CNS. Astrocytes are cells with multiple functions, and they play major roles in regulating neurons and responding to exogenous pathogens through the expression of various factors and have a supportive effect on nerve tissue in the CNS. Therefore, viral infection of astrocytes could affect nervous system homeostasis in various ways, especially the homeostasis of brain stem neurons. The up-regulation of noradrenalin, dopamine and IL-6 secretion from infected astrocytes suggested the direct stimulation of neurons. Noradrenalin is an excitatory neurotransmitter that is capable of inducing excitatory signal transduction in the hypothalamic-pituitary-adrenal (HPA) pathway, which usually promotes adrenalin up-regulation in the peripheral blood and induces abnormally high blood pressure (Mastorakos et al., 1993; Chrousos, 1995). Obviously, this pathophysiological process could be recognized as an important reason for neurogenic pulmonary edema and subsequent cardiopulmonary failure. Additionally, up-regulated IL-6 modulates the signal transduction of paraventricular nuclei of the thalamus in a neuroendocrine-immune system and activates downstream neural pathways (Wang et al., 2012), also suggesting the stimulation of neurons by infected astrocytes. In this study, an up-regulation in the secretion of adrenalin, another excitatory neurotransmitter, was observed in neurons co-cultured with the infected astrocytes in the co-culture system. This suggested a response from the neurons stimulated by infected astrocytes via IL-6 and noradrenalin, which implied a combined effect of noradrenalin and IL-6 that induced an over-excitatory polarization in the neural net in brain tissue after EV71 entry into the CNS. In this case, the pathogenesis of neurogenic pulmonary edema in severe cases of EV71-infected HFMD might be understandable. However, our further detection of adrenalin in the peripheral blood of infected macaques indicated that this neurotransmitter was up-regulated (Figure 2I).

All of the data described here and in our previous study provide a conceptual framework for the pathogenesis of EV71 CNS infection. In contrast to the pathological characteristics of other viral encephalitis cases, EV71 infection in the CNS involves astrocytes and exhibits a mechanism of viral pathogenesis in which the homeostasis of the nervous system is disrupted by variations of production of the neurotransmitters including noradrenalin and adrenalin in neurons modulated by the infected astrocytes. These effects lead to the clinical manifestation of neurogenic pulmonary and cardiopulmonary failure. Further evidence from clinical patients is needed to support this conclusion.

## Author contributions

Conceived and designed the experiments: QL and MF; performed the experiments: MF, SF, SG, YZ, XZ, YL, LW, JBW, JJW, and NM; analyzed the data: MF, SF, XZ, YZ, QL, YC, and LL; contributed reagents/materials/analysis tools: XZ, TZ, JJW, and LY; contributed to the writing of the manuscript: QL, MF, YZ, XZ, and YC.

### Conflict of interest statement

The authors declare that the research was conducted in the absence of any commercial or financial relationships that could be construed as a potential conflict of interest.
