# Supplementary material for: The Preferential Infection of Astrocytes by Enterovirus 71 Plays a Key Role in the Viral Neurogenic Pathogenesis
Source: Front Cell Infect Microbiol. 2016 Dec 21;6:192. doi: 10.3389/fcimb.2016.00192 (PMC5174126; doi:10.3389/fcimb.2016.00192)
Supplement: Supplementary file 1 [file DataSheet1.pdf]

Supplementary

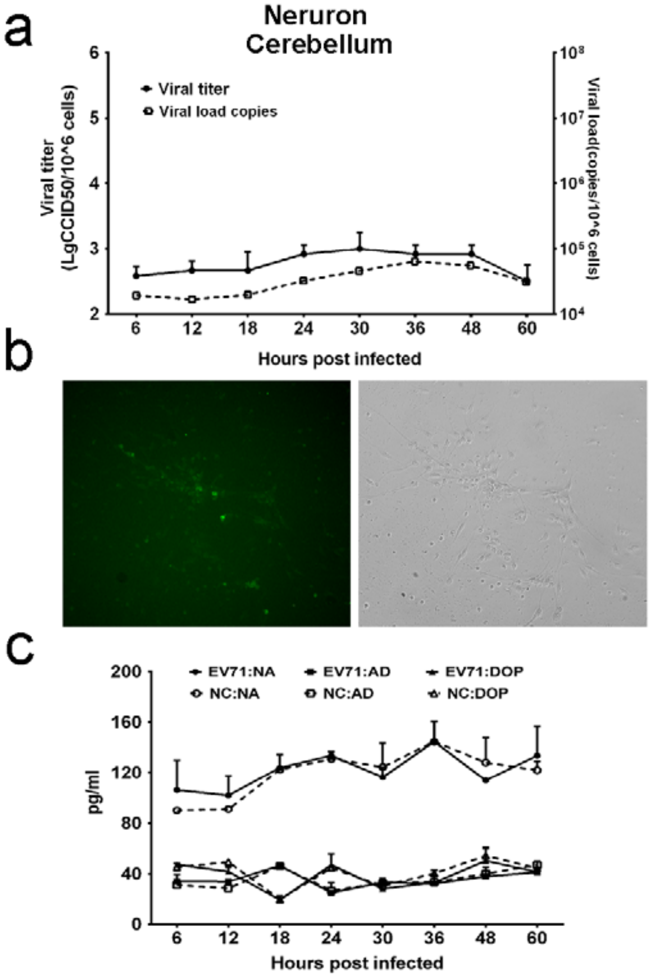

S1. Detection of EV71 replication in cultured neurons from the cerebellum *in vitro*.

Proliferation of the virus in cultured human neurons (a) was measured based on virus titration and the viral load from 6 to 60 hours post infection. Immunofluorescence microscopy observations of the EV71 antigen in cultured EV71-infected neurons (b, images are shown at 100 × magnification). Monoamine release by neurons (c) from 6 to 60 hours post EV71 infection. NA: noradrenaline; AD: adrenaline; DOP: dopamine.

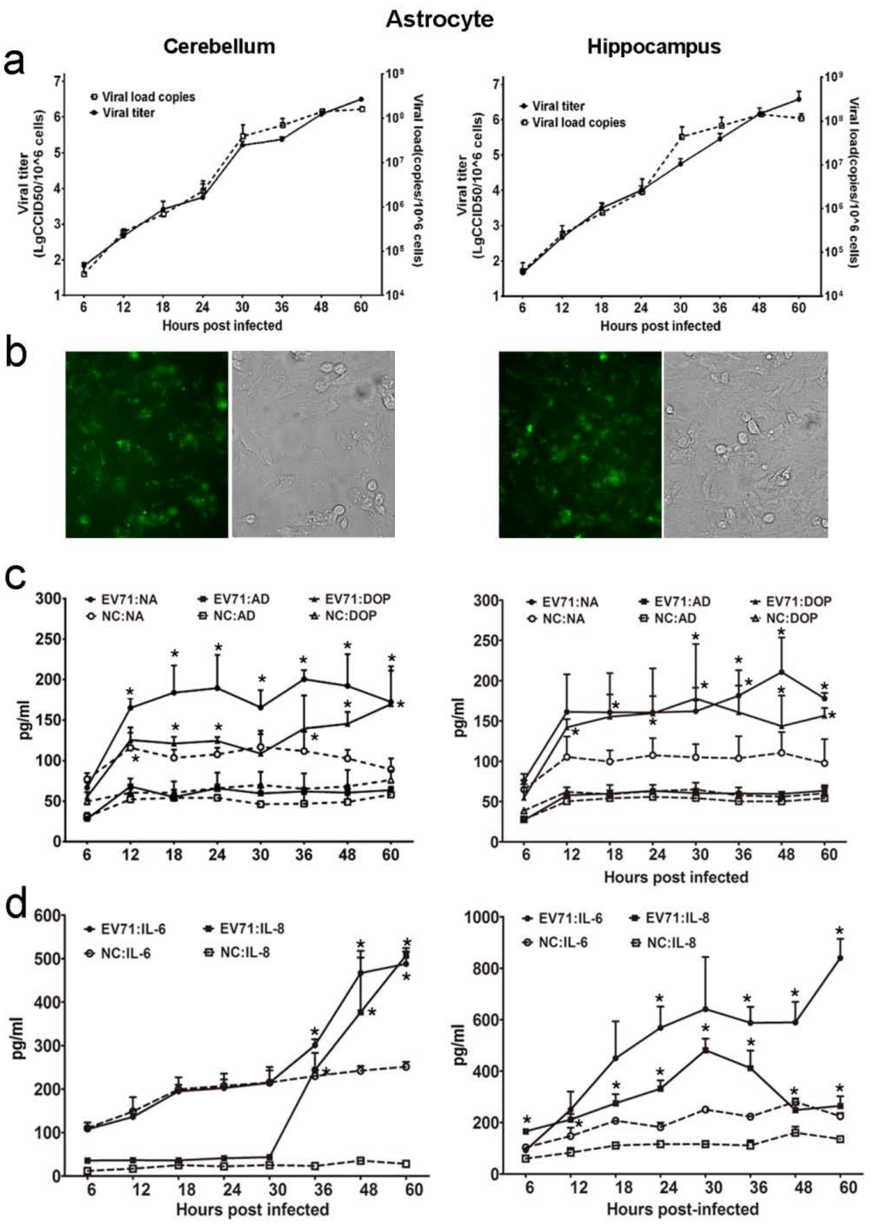

S2. Detection of EV71 replication in cultured astrocytes from the cerebellum and hippocampus *in vitro*.

Proliferation of the virus in cultured human astrocytes (a) was measured based on virus titration and the viral load from 6 to 60 hours post infection. Immunofluorescence microscopy observations of the EV71 antigen in cultured EV71-infected astrocytes (b, images are shown at 400 × magnification). Monoamine (c) and cytokines (d) release by astrocytes from 6 to 60 hours post EV71 infection. NA: noradrenaline; AD: adrenaline; DOP: dopamine.*$p \leq 0.05$, compared with the corresponding control group.

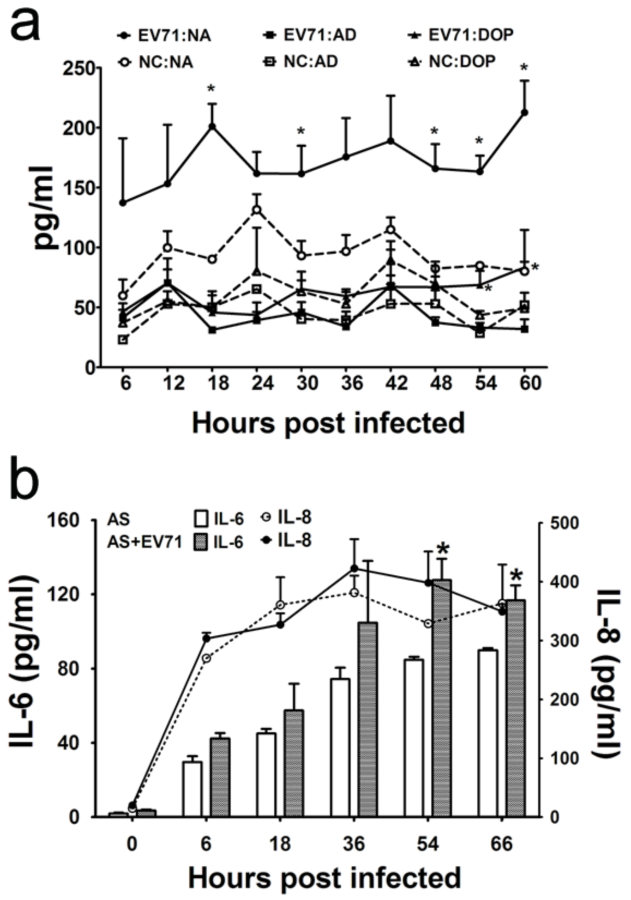

S3. Monoamine (a) and cytokines (b) detected in rhesus macaque astrocytes with EV71 infection. NA: noradrenaline; AD: adrenaline; DOP: dopamine; NC: negative control. *$p \leq 0.05$ compared to the corresponding control group.

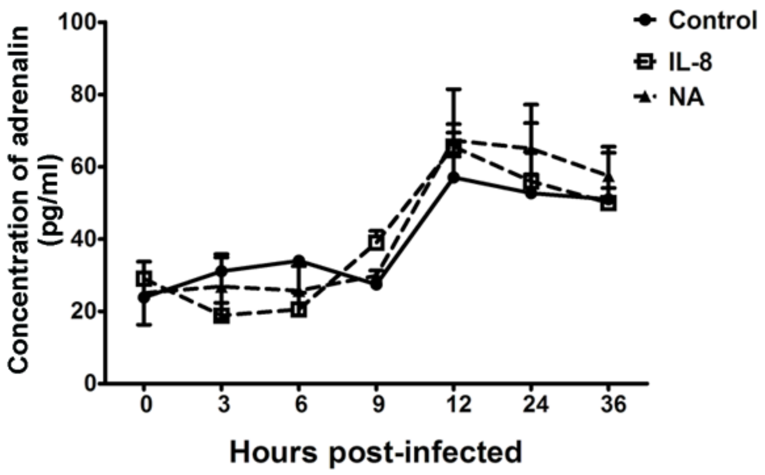

S4. Adrenaline released by neurons from brain stem treated with IL-8 and noradrenalin. NA: noradrenaline

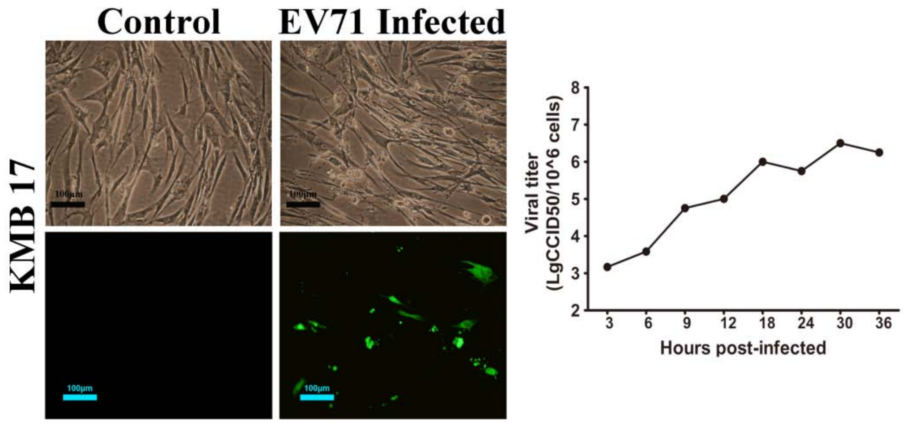

S5. EV71 infected human fibroblasts in vitro. (a) The cytopathic effect of KMB 17 cells 24 hours post EV71 infection , as observed by microscopy. Immunofluorescence confocal microscopy observations of KMB 17 infected with EV71 using fluorescent anti-EV71 antibodies. (b) The proliferation of virus in KMB 17 cells, and measured by virus titration from 3-36 hours p.i. (MOI=0.05).
